# Least mean square fourth based microgrid state estimation algorithm using the internet of things technology

**DOI:** 10.1371/journal.pone.0176099

**Published:** 2017-05-01

**Authors:** Md Masud Rana

**Affiliations:** Department of Electronics and Communication Eegineering, Khulna University of Engineering and Technology, Khulna, Bangladesh; Chongqing University, CHINA

## Abstract

This paper proposes an innovative internet of things (IoT) based communication framework for monitoring microgrid under the condition of packet dropouts in measurements. First of all, the microgrid incorporating the renewable distributed energy resources is represented by a state-space model. The IoT embedded wireless sensor network is adopted to sense the system states. Afterwards, the information is transmitted to the energy management system using the communication network. Finally, the least mean square fourth algorithm is explored for estimating the system states. The effectiveness of the developed approach is verified through numerical simulations.

## 1 Introduction

Due to environmental consciousness and energy crisis, the distributed energy resources (DERs) such as solar panels are integrated into the grid. As their generation pattern is intermittent in nature, it is required to monitor them closely. In order to monitor them, the internet of things (IoT) is the potential infrastructure as it provides the endless support and wide-area connectivity. The main technologies for the IoT are machine-to-machine interfaces, wireless sensor network (WSN) and embedded sensor and actuator schemes that facilitates sensing, control interaction as well as networking [1]. Obviously, an unreliable communication network exits between sensors and energy management system (EMS). The function of the EMS is to estimate the system states and maintain the grid stability. Driven by these motivations, this paper proposes a least mean square based state estimation algorithm considering the packet dropouts in the measurement under the umbrella of the IoT framework.

Extensive investigations on power system state estimations have been carried out in the literature. To begin with, the least mean squares (LMS) based power system dynamic state estimation is explored in [2, 3]. Unfortunately, it is very difficult to tune the step size parameter of this LMS scheme. The normalized LMS (NLMS) algorithm is proposed for state estimations [2, 4]. This NLMS process normalises the input signal so that the step size parameter can guarantee the stability of this algorithm. Afterwards, the variable step size based LMS algorithm for system state estimation is proposed in [5, 6]. This approach provides an optimum convergence rate but there is a trade-off between rate and performance. An artificial intelligence based dynamic state estimation is presented in [7]. This technique does not require a mathematical model. However, in stability analysis of large-scale power systems, it is often preferable to have an exact model. From this perspective, the physical model based Kalman filter (KF) algorithm is widely used for dynamic state estimations [8–10]. Unfortunately, the computational complexity is extensive compared with the LMS algorithm. A semidefinite programming based optimal energy management system is proposed in [11]. The smart home is designed considering the photovoltaic systems and energy storage with their uncertainties. Finally, a semidefinite programming problem is formulated to optimize the electric power allocation in the battery, home load demand, photovoltaic power supply and utility grid. All of the aforementioned papers assumed that the communication is perfect between sensors and EMS. In reality, there are a lossy communication channels between them. The main contributions of this paper are summarized as follows:

- The considered environmentally-friendly renewable microgrid is expressed as a state-space model. Then the IoT based wireless sensor network is adopted to transfer the sensing state information to the energy management system where the measurements are lost over unreliable communication channels. For long distance data transmission, the IoT based digital communication scheme is proposed.
- The least mean square fourth algorithm is proposed for estimating the system states over communication channels. The fourth error function is minimized with respect to the predicted states which leads to a new estimation algorithm in the context of the IoT based smart grid communication.

The rest of the article is organised as follows. In Section 2 presents the microgrid state-space model, and its estimation algorithm is in Section 3. The simulation results and conclusion are in Section 4 and 5, respectively.

**Notation:** Bold face lower and upper case letters are used to represent vectors and matrices, respectively; superscripts **x**′ denotes the transpose of **x** and **I** is the identity matrix.

## 2 Modelling the microgrid

**Fig 1:**
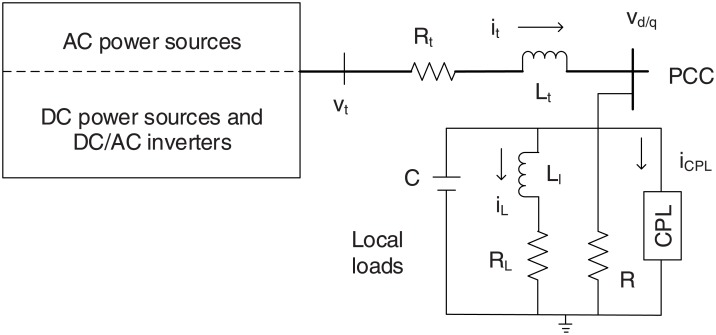
The schematic diagram of a microgrid [12, 13].

One of the vital characteristics of the smart grid is to integrate the renewable resources into the electricity network. To demonstrate, the schematic diagram of an isolated studied microgrid is illustrated in Fig 1 [12–14]. It can be observed that the microgrid is connected with the grid/another microgrid through the point common coupling points (PCCs). Basically, the energy generating unit of a microgrid entails of AC/DC voltage sources, DC/AC converter and series RLC filter. The load is connected to the PCC, and the local loads are equivalent to resistive, capacitive and constant power load (CPL). After applying Kirchhoff’s voltage and Kirchhoff’s current laws, the dynamic of a microgrid in the dq-frame is expressed as follows [12–14]:

$$\begin{array}{c} {\dot{i}}_{td}=\left(-R_{t}i_{td}+\omega L_{t}i_{tq}-v_{d}+v_{td}\right)/L_{t}. \end{array}$$ (1)

$$\begin{array}{c} {\dot{i}}_{tq}=\left(-R_{t}i_{tq}-\omega L_{t}i_{td}-v_{q}+v_{tq}\right)/L_{t}. \end{array}$$ (2)

$$\begin{array}{c} {\dot{i}}_{Ld}=\left(-R_{L}i_{Ld}+\omega L_{l}i_{Lq}+v_{d}\right)/L_{l}. \end{array}$$ (3)

$$\begin{array}{c} {\dot{i}}_{Lq}=\left(-R_{L}i_{Lq}-\omega L_{l}i_{Ld}+v_{q}\right)/L_{l}. \end{array}$$ (4)

$$\begin{array}{c} {\dot{v}}_{d}=\left(i_{td}-i_{Ld}-v_{d}/R+\omega Cv_{q}-i_{CPLd}\right)/C. \end{array}$$ (5)

$$\begin{array}{c} {\dot{v}}_{q}=\left(i_{tq}-i_{Lq}-v_{q}/R+\omega Cv_{d}-i_{CPLq}\right)/C. \end{array}$$ (6)

Here, *i*, R, L, C, *ω*, *v*_*d*_, and *v*_*t*_ are the current, resistor, inductor, capacitor, frequency, PCC voltage and line voltage, respectively. For instance, *i*_*td*_ and *i*_*tq*_ represent the line current in the d-q frame.

**Fig 2:**
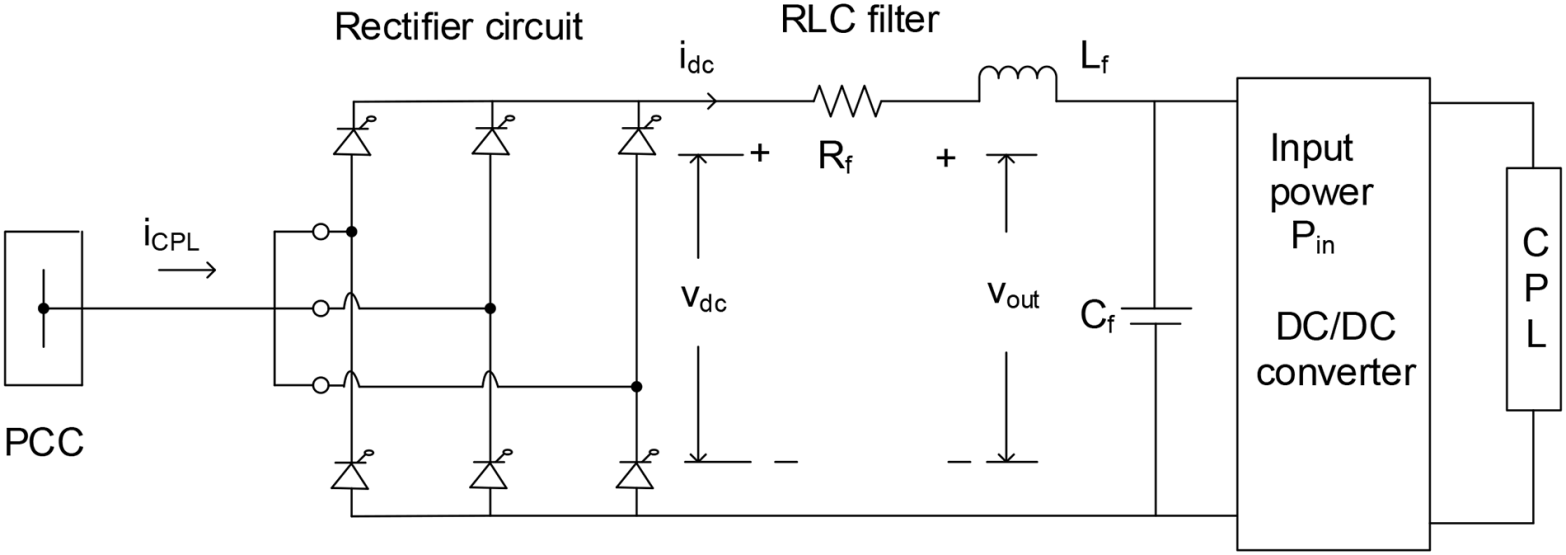
3-phase diode rectifier and RLC filter in an AC microgrid [12, 13].

Usually, the CPL is connected to the AC bus via the rectifier circuit, RLC filter and converter. To clarify, Fig 2 shows the schematic diagram of a CPL in the considered microgrid. The dynamic equation of RLC dc-link filter is expressed as follows:

$$\begin{array}{c} {\dot{i}}_{dc}=\left(v_{dc}-R_{f}i_{dc}-v_{out}\right)/L_{f}. \end{array}$$ (7)

$$\begin{array}{c} {\dot{v}}_{out}=\left(i_{dc}-\frac{P_{in}}{v_{out}}\right)/C_{f}. \end{array}$$ (8)

Here, *i*, *P*_*in*_, v, L, and C represent the maximum current, converter/inverter input power, peak voltage, inductor and capacitor, respectively. For example, *i*_*dc*_ and *v*_*out*_ are the RLC filter current and output voltage, respectively. With appropriate firing angle and switching function in the rectifier circuit, the CPL line current *i*_*CPLd*_ for Eq (5) is given by [15]:

$$\begin{array}{c} i_{CPLd}=S_{w}i_{dc}=\sqrt{\frac{3}{2}}\frac{2\sqrt{3}}{\pi }i_{dc}. \end{array}$$ (9)

$$\begin{array}{c} i_{CPLq}=0. \end{array}$$ (10)

$$\begin{array}{c} v_{dc}=S_{w}v_{d}=\sqrt{\frac{3}{2}}\frac{2\sqrt{3}}{\pi }v_{d}. \end{array}$$ (11)

Here, the maximum magnitude of the stitching function *S*_*w*_ is equal to $\sqrt{\frac{3}{2}}\frac{2\sqrt{3}}{\pi }$ [13].

Now the above nonlinear set of system equations is linearised around the operating point *x*^⋆^ i.e., $x_{i}-x_{i}^{⋆}$ where $x_{i}^{⋆}$ is the operating point [12, 13]. After simple algebraic manipulations, the continuous-time state-space model of a microgrid is expressed as follows:

$$\begin{array}{cc} \dot{x} & =A_{c}x+B_{c}u+n, \end{array}$$ (12)

where **x** = [Δ*i_td_* Δ*i_tq_* Δ*i_Ld_* Δ*i_Lq_* Δ*v_d_* Δ*v_q_* Δ*i_dc_* Δ*v_out_*], $A_{c}=[\begin{array}{cccccccc} \frac{-R_{t}}{L_{t}}\; & \omega & 0 & 0 & \;\frac{-1}{L_{lt}} & 0 & 0 & 0\\ -\omega & \;\frac{-R_{t}}{L_{t}}\; & 0 & 0 & 0 & \;\frac{-1}{L_{t}}\; & 0 & 0\\ 0 & 0 & \frac{-R_{L}}{L_{l}} & \omega & \frac{1}{L_{l}} & 0 & 0 & 0\\ 0 & 0 & -\omega & \frac{-R_{L}}{L_{l}} & 0 & \frac{1}{L_{l}} & 0 & 0\\ \;\frac{1}{C}\; & 0 & \;\frac{-1}{C}\; & 0 & \;\frac{-1}{RC}\; & \omega & -\frac{S_{w}}{C} & 0\\ 0 & \;\frac{1}{C}\; & 0 & -\;\frac{1}{C}\; & -\omega & \;\frac{-1}{RC}\; & 0 & 0\\ 0 & 0 & 0 & 0 & \frac{S_{w}}{L_{f}} & 0 & \;-\frac{R_{f}}{L_{f}}\; & \;\frac{-1}{L_{f}}\;\\ 0 & 0 & 0 & 0 & 0 & 0 & \frac{1}{C_{f}} & \frac{P_{in}}{C_{f}v_{out}^{2⋆}} \end{array}]$, $S_{w}=\sqrt{\frac{3}{2}}\frac{2\sqrt{3}}{\pi }$, $B_{c}={\left[0\;0\;0\;0\;0\;0\;0-1/\left(v_{out}^{⋆}C_{f}\right)\right]}^{\prime }$ and *u* = *δP*_*in*_. The symbol **n** is the process uncertainty. The process noise **n** is the zero mean Gaussian distribution [16–18] whose covariance matrix is **Q**. The system is expressed as a disrate-time state-space model as follows:

x(k+1)=Ax(k)+Bu(k)+n(k), (13)

where **A** = **I** + Δ*_t_***A***_c_*, **B** = Δ*_t_***B***_c_*, and Δ_*t*_ is step size parameter.

**Fig 3:**
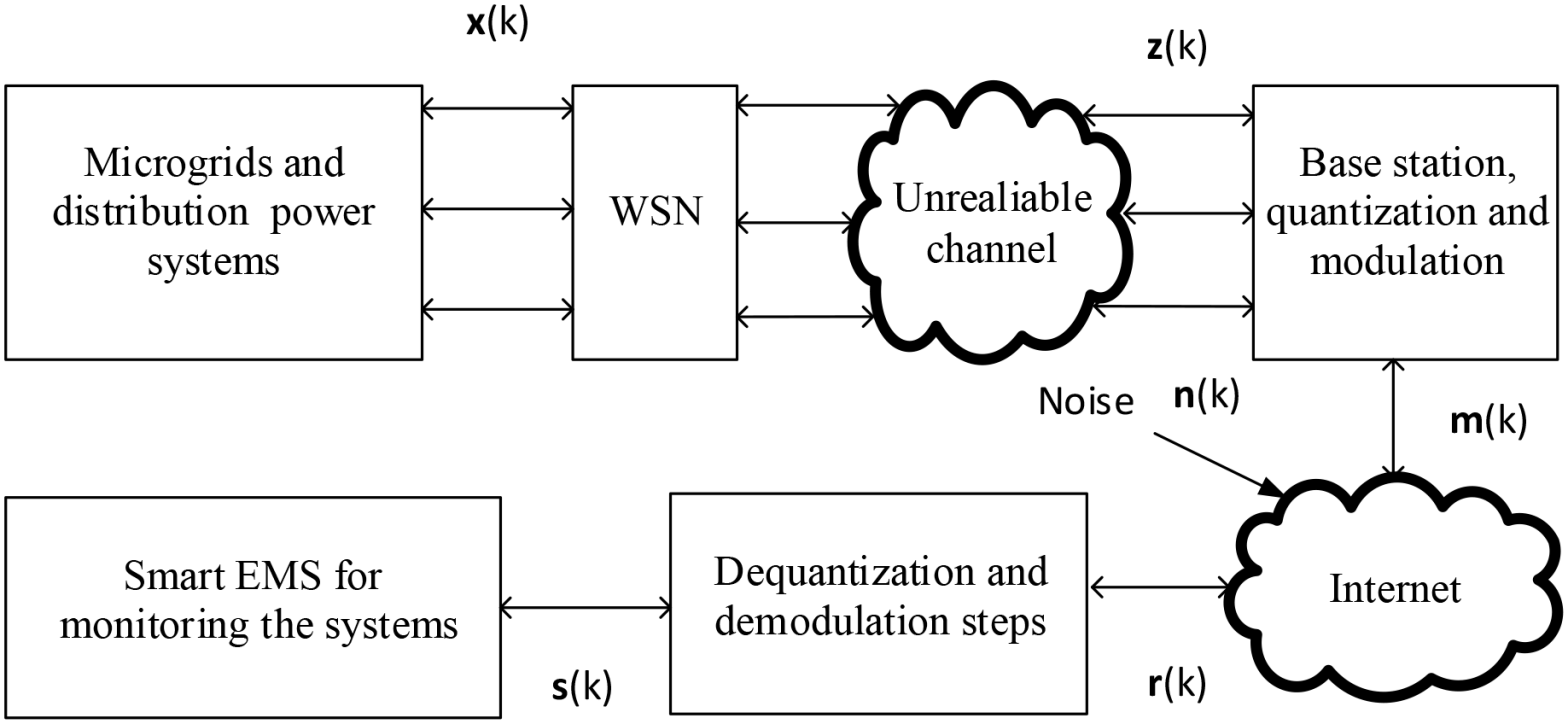
Proposed architecture.

In order to monitor the microgrid farm, the service provider deploys the IoT elements such as sensors around the microgrids. The IoT infrastructure for sensing and estimating the system state is illustrated in Fig 3 [19, 20]. Basically, the voltage and current sensors are deployed to measure the microgrid states. Then it transmits to the energy management system through the internet which adds noise. The received signal at the energy management system is given by:

z(k)=λ(k)Hx(k)+λ(k)w(k), (14)

where **C**(*k*) is the observation matrix, **w**(*k*) is the noise which considers zero mean Gaussian distribution with **R**(*k*) covariance matrix, *λ*(*k*) is the packet loss parameter with

$$\begin{array}{c} \lambda \left(k\right)=\left\{\begin{array}{cc} 1, & \;\;\text{no}\;\text{loss,}\;\\ \gamma (k),\;\;\;0<\gamma (k)<1 & \;\;\text{lossy}\;\text{condition}.\; \end{array}\right. \end{array}$$

Here, *γ*(*k*) is the packet arrival probability [21–23].

In order to transmit the sensing information to the EMS, the sensing information is quantized to obtain the bit information [19, 20]. This is due to the fact that the digital modulation scheme is used for long distance signal transmission. So, the bit information is modulated using the binary phase shift keying (BPSK) to obtain the modulated signal **m**(*k*). The modulated signal is then transmitted through the internet, which causes the signal distortions. The received signal at the control center is written as follows.

$$\begin{array}{c} r(k)=m(k)+n(k), \end{array}$$ (15)

where **n**(*k*) is the channel noise.

## 3 Proposed estimation algorithm

According to the steepest decent approach, the state updated rule is given by [2, 3, 24]:

$$\hat{x}\left(k+1\right)=\hat{x}\left(k\right)-\mu \Delta J\left(k\right).$$ (16)

Here, $\hat{x}\left(k\right)=A\hat{x}\left(k-1\right)+Bu\left(k\right)$ is the predicted states, $\hat{x}\left(k-1\right)$ is the previous estimated states, *μ* is the scalar step size parameter, *J*(*k*) is the objective function to be minimized and Δ**J**(*k*) is the gradient decent. The cost function is defined by [2, 3]:

$$J\left(k\right)=E\left[\overset{m}{\underset{n=1}{\sum }}e_{n}^{4}\left(k\right)\right].$$ (17)

Here, **e**_*n*_(*k*) is the n-th error function, which is defined as follows:

$$e_{n}\left(k\right)=z_{n}\left(k\right)-{\hat{z}}_{n}\left(k\right).$$ (18)

Here, ${\hat{z}}_{n}\left(k\right)=\lambda \left(k\right)H_{n}\hat{x}\left(k\right)$ and **H**_*n*_ is the n-th row of the observation matrix. The minimization of the cost function with respect to the predicted states lead to the following expression:

$$\Delta J\left(k\right)=-4\lambda \left(k\right)\sum_{n=1}^{m}e_{n}^{3}\left(k\right)H_{n}^{\prime }.$$ (19)

From Eq (16) and Eq (19), the update state estimation is expressed by [2, 3]:

$$\hat{x}\left(k+1\right)=\hat{x}\left(k\right)+\mu \lambda \left(k\right)K\sum_{n=1}^{m}e_{n}^{3}\left(k\right)H_{n}^{\prime }.$$ (20)

Here, **K** is the user defined matrix, which depends on the specific application [2, 3]. In order to obtain the consistent estimation performance, the following normalization version is proposed:

$$\hat{x}\left(k+1\right)=\hat{x}\left(k\right)+\frac{\mu \lambda \left(k\right)K\sum_{n=1}^{m}e_{n}^{3}\left(k\right)H_{n}^{\prime }}{\epsilon +{\left|\left|K\right|\right|}_{F}^{4}}.$$ (21)

Here, *ϵ* is the small positive value to avoid division by zero [25]. It can be seen that the proposed least mean square fourth algorithm is very simple, and it does not need to know the observation noise covariance as opposed to the KF scheme. When the communication is involved then $\hat{z}\left(k\right)$ is equivalently replaced by the dequantized output **s**_*n*_(*k*) i.e., ${\hat{z}}_{n}\left(k\right)\approx s_{n}\left(k\right)$. This is due to the fact that the depending on the designed communication scheme, the infrastructure is equivalently reconstructed ${\hat{z}}_{n}\left(k\right)$ which is also considered by different application domain papers in [26, 27].

## 4 Numerical simulation results

The effectiveness of the developed approach is verified through the numerical simulation. After modelling the microgrid, the developed scheme is applied to the microgrid state estimations. To do this, the simulation parameters are described in Table 1 [12, 13], where Matlab is used as a simulator. The process and measurement noises are considered as zero mean white Gaussian noises with variances **Q** = 0.00001**I** and **R** = 0.00012**I**, respectively. The considered value of **K** is 0.16 × *eye*(8). The sampling step size parameter is 0.000001. The system is considered as a balanced load with unregulated microgrid.

**Table 1:** AC microgrid and its parameters.

Symbols	Values	Parameters	Values
R_*L*_	5 Ohm	R_*t*_	1.5 × 10^−3^ Ohm
R_*l*_	50 × 10^−3^ Ohm	R_*f*_	76 Ohm
*L*_*t*_	70 × 10^−6^ *H*	*L*_*l*_	111.9 × 10^−3^ *H*
*C*	62.855 × 10^−6^ *F*	*C*_*f*_	1 × 10^−3^ *F*
*P*_*in*_	0.7 pu W	*γ*	0.95
*μ*	0.15	*ϵ*	0.00019

**Fig 4:**
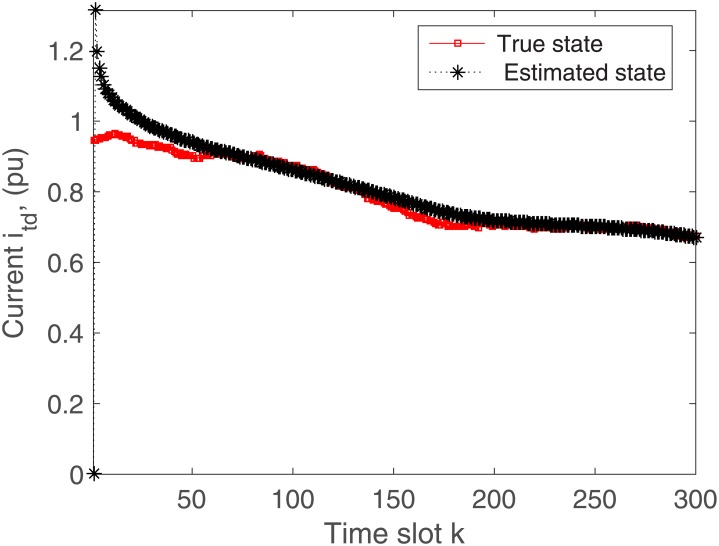
Actual versus estimated values of *i*_*td*_ without delays.

**Fig 5:**
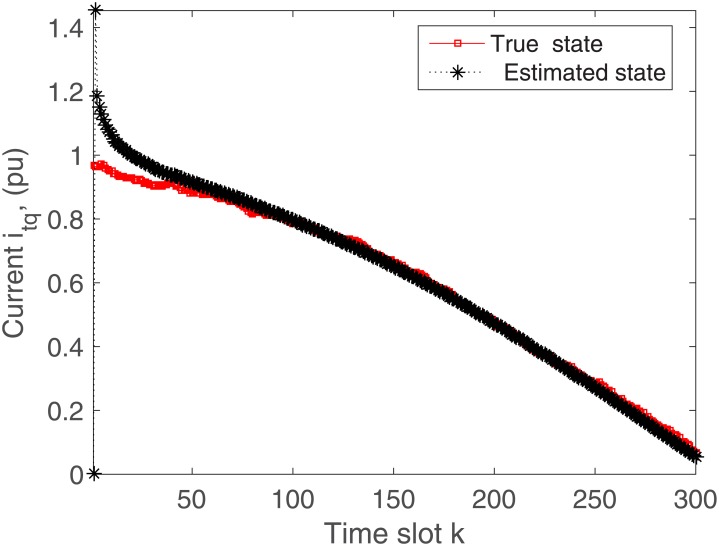
Actual versus estimated values of *i*_*tq*_ without delays.

**Fig 6:**
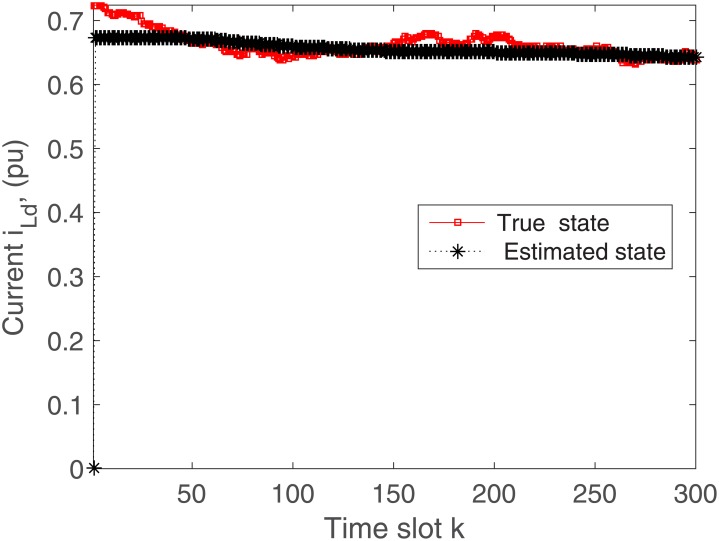
Actual versus estimated values of *i*_*Ld*_ without delays.

**Fig 7:**
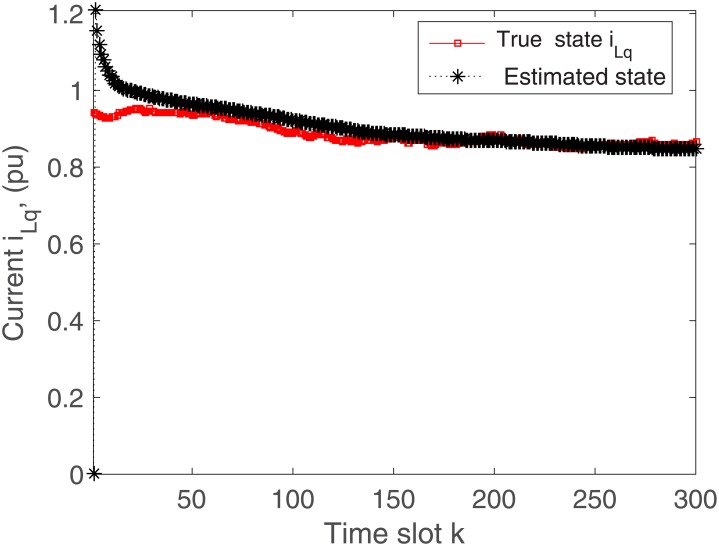
Actual versus estimated values of *i*_*Lq*_ without delays.

**Fig 8:**
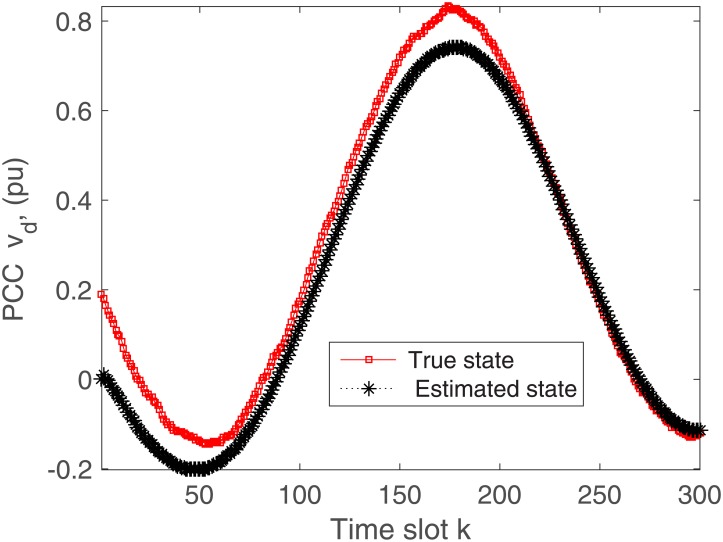
Actual versus estimated values of *v*_*d*_ without delays.

**Fig 9:**
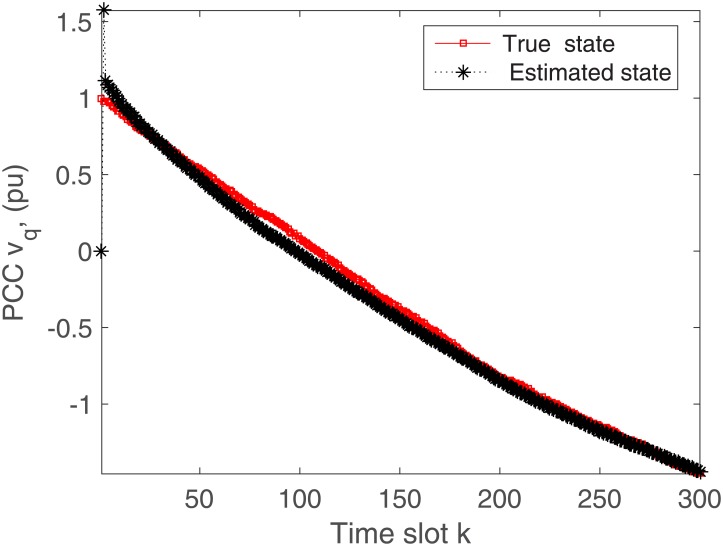
Actual versus estimated values of *v*_*q*_ without delays.

The proposed algorithm is tested on the microgrid with and without communication delays. The simulation results without delays are presented in Figs 4–9. First of all, Figs 4 and 5 show the direct-quadrature (dq) axis filter currents versus time slots. It can be seen that the proposed scheme is able to estimate these states within 0.00015-0.0002 seconds (time slot × step size = 200 × 0.000001). This is due to the fact that the developed approach is able to perfectly rectify the estimation errors as time proceeds. Moreover, the simulation results in Figs 6 and 7 illustrate the dq-frame load currents versus time. It can be observed that the actual states are matched with the estimated states within a short time. Furthermore, the point of common coupling voltages in the dq-frame are illustrated in Figs 8 and 9. Even if the common coupling voltages are changed with the local loads and renewable energy generation patterns, but the proposed algorithm can effectively track the system states. Other microgrid state responses have similar kind of estimation performance. Note the small fluctuations due to the Gaussian noises and packet losses.

**Fig 10:**
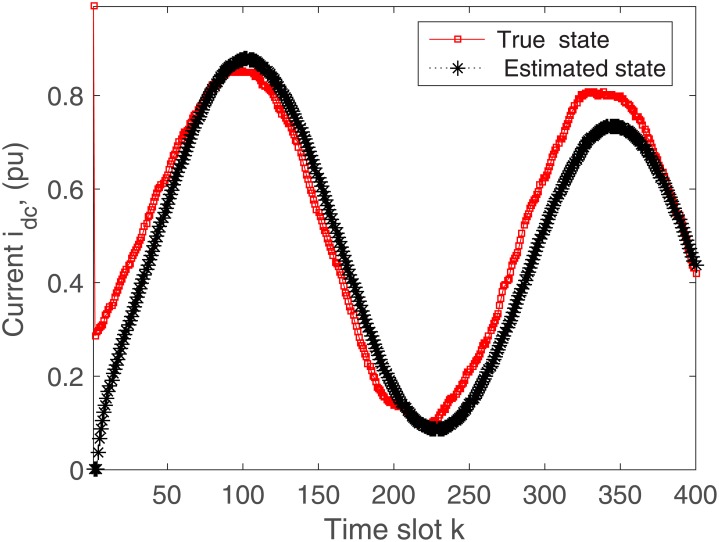
Actual versus estimated values of *i*_*dc*_ with delays.

**Fig 11:**
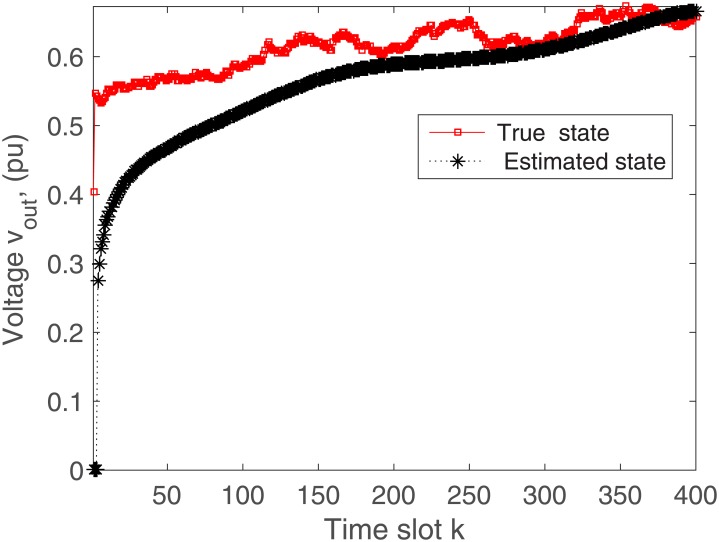
Actual versus estimated values of *v*_*out*_ with delays.

In practical situation, there may be the communication delays when sensing the system states. Considering 2 sample communication delays in the observation, the rectifier output current and voltage are demonstrated in Figs 10 and 11, respectively. It is clearly seen that the actual system states match the estimated states within 0.0004 seconds. As the estimation algorithm is designed without communication delays, so the proposed scheme will need more time to estimate the microgrid states as expected.

## 5 Conclusion and future work

This paper proposes a least mean square fourth algorithm considering the packet dropouts. First of all, the microgrid incorporating the renewable distributed energy resources is represented by a state-space model. Then the innovative IoT infrastructure is proposed for transferring the microgrid information to the energy management system for estimating the system states. The effectiveness of the developed approach is verified through numerical simulations. It can be seen that the developed approach is very effective in estimating the system states. The future work involves applying the suitable control algorithm so that the system will be stabilized.
